# Lipidomic Profile of Individuals Infected by *Schistosoma mansoni*

**DOI:** 10.3390/ijms26157491

**Published:** 2025-08-02

**Authors:** Thainá Rodrigues de Souza Fialho, Ronald Alves dos Santos, Yuri Tabajara, Ane Caroline Casaes, Michael Nascimento Macedo, Bruna Oliveira Lopes Souza, Kelvin Edson Marques de Jesus, Leonardo Paiva Farias, Camilla Almeida Menezes, Isadora Cristina de Siqueira, Carlos Arterio Sorgi, Adriano Queiroz, Ricardo Riccio Oliveira

**Affiliations:** 1 Instituto Gonçalo Moniz, Fundação Oswaldo Cruz, Fiocruz Bahia, Salvador 40296-710, Brazil; thaina.fialho@outlook.com (T.R.d.S.F.); ronald.santos@fiocruz.br (R.A.d.S.); yuritabajara@gmail.com (Y.T.); ane.casaes@fiocruz.br (A.C.C.); macedo.m@outlook.com (M.N.M.); bruna.souza@fiocruz.br (B.O.L.S.); kelvinedson2@gmail.com (K.E.M.d.J.); leonardo.farias@fiocruz.br (L.P.F.); camilla.almeida@fiocruz.br (C.A.M.); isadora.siqueira@fiocruz.br (I.C.d.S.); adrianoqs@gmail.com (A.Q.); 2 Faculdade de Ciências Farmacêuticas de Ribeirão Preto, Universidade de São Paulo—USP, São Paulo 01246-904, Brazil; carlos.sorgi@usp.br

**Keywords:** *Schistosoma mansoni*, lipidomics, schistosomiasis, lipid profile

## Abstract

*Schistosoma mansoni* infection is associated with hepatic inflammation and fibrosis, but its systemic metabolic effects remain poorly understood. This study aimed to investigate changes in the serum lipidomic profile associated with *S. mansoni* infection and parasite load in individuals from an endemic area. This cross-sectional analysis was nested within a longitudinal cohort study conducted in northeastern Brazil. Parasitological diagnosis and quantification were performed using the Kato–Katz technique. A total of 45 individuals were selected and divided into three groups: high parasite load (HL), low parasite load (LL), and uninfected controls (NegE). Serum samples were analyzed using mass-spectrometry-based lipidomics. The most abundant lipid subclasses across all groups were phosphatidylcholines (PC), triacylglycerols (TAG), and phosphatidylethanolamines (PE). However, individuals in the HL group exhibited distinct lipidomic profiles, with increased levels of specific phosphatidylinositols (PI) and reduced levels of certain TAG species compared to the NegE group. These changes may reflect host–parasite interactions and immune–metabolic alterations driven by intense infection. Our findings suggest that *S. mansoni* infection, particularly at higher parasite burdens, can influence the host’s serum lipid profile and may contribute to metabolic disturbances in endemic populations.

## 1. Introduction

Human schistosomiasis affects approximately 240 million people, with almost 700 million living in areas at risk. This is a tropical infection that has undergone epidemiological changes in recent decades. The disease is no longer present only in rural regions but has begun to show a prevalence of cases in urban areas, mainly those with deficiencies in basic sanitation [1,2,3]. Some regions remain affected by the presence of transmitting mollusks; it is estimated that about 1.5 million people still live in at-risk areas in Brazil [4]. *Schistosoma mansoni* is the etiological agent of the disease, with humans as its main host and freshwater snails of the genus *Biomphalaria* as its intermediate host. Of the six species that parasitize humans, *S. mansoni* stands out in Latin America [3,5].

Two clinical forms of the disease are known: the acute and the chronic forms, which induce different clinical manifestations. In the acute phase, urticarial eruptions may occur, which are caused by the penetration of cercariae into the skin of individuals. However, in endemic areas, this dermatitis is not recognized. Clinical manifestations can vary from individual to individual, including high fever, diarrhea, nausea/vomiting, edema, and urticaria. The main lesions arising during the chronic phase are caused by eggs that secrete proteolytic enzymes that provoke inflammatory and eosinophilic granulomatous reactions, affecting organs such as the liver, spleen, and lungs, which are progressively replaced by fibrosis. The chronic phase can present two manifestations forms: the intestinal or hepatointestinal and the hepatosplenic [3,6,7].

Some studies suggest that schistosomiasis may lead to alterations in lipid metabolism in humans since disturbances in protein synthesis and the various interleukins released during inflammation can affect lipoprotein metabolism [8]. Dyslipidemia in the severe form of the infection has been associated with increased levels of phospholipids and reduced levels of cholesterol ester [9]. A study on the metabolic response to *S. japonicum* infection in mice observed lower levels of glucose and glycogen, accompanied by higher levels of choline metabolites—such as phosphorylcholine (PC) and glyceryl phosphorylcholine (GPC)—as well as alanine, compared to controls prior to sexual maturation [10]. In addition, significantly decreased levels of triacylglycerol and cholesterol esters were also demonstrated in the liver and ileum in mice with the progression of infection by *S. mansoni* [11]. Another study also reported that infected mice reduced the hepatic expression of acetyl coenzyme A acyltransferase, which is an enzyme directly involved in the metabolism of fatty acids [11,12,13,14]. Additionally, one study observed alterations in lecithin-cholesterol acyltransferase (LCAT), an enzyme that plays an important role in the normal metabolism of lipoproteins and is responsible for the formation of almost all esterified cholesterol. It has been demonstrated that in some cases, people infected with *S. mansoni* have decreased LCAT activity, thus reducing the levels of total cholesterol [15,16,17].

Here, we investigated the serum lipid profile of individuals naturally exposed to *Schistosoma mansoni* in an endemic area, stratified by high and low parasitic loads, alongside uninfected controls. Our aim was to explore lipidomic signatures associated with *S. mansoni* infection and parasite burden under real-life conditions. Our findings revealed PC, HexCer, and FFA as the most abundant lipid subclasses in patients with schistosomiasis. A positive correlation was observed between parasitic load and gamma-glutamyltransferase (GGT) levels. The results presented herein may be valuable for the identification of biomarkers of evolution to severe disease.

## 2. Results

The average age of all participants included in D0 was 28 ± 18 years, of which 194 (57.06%) were women. Regarding household characteristics, 99.15% of residents had piped water, and approximately 63% used septic tanks. Furthermore, the family income of 84.62% of the study participants was less than one minimum wage during the interview period (2018), as shown in Table 1.

Table 1 also presents the hematological profile of individuals. The median hemoglobin was 12.70 (11.80–13.68) g/dL, the median leukocyte count was 6710 (5223–8205)/µL, and the platelet count was 268 (224–320) thousand/µL. The analysis of the hepatic profile showed that the median AST was 22.37 (18.64–32.63) U/L, the median ALT was 12 (9–18) U/L, and the median GGT was 23.66 (17.21–40.63) U/L.

The prevalence of infections and parasite loads of the helminths *Schistosoma mansoni*, *Ascaris lumbricoides*, and *Trichuris trichiura* were determined using the Kato–Katz method. In 55.59% (n = 189) of the participants, *S. mansoni* eggs were identified in at least one of the two slides. For the other helminths, *Ascaris lumbricoides* infection was observed at 40.29% (n = 137), and *Trichuris trichiura* in 53.82% (n = 183) of the participants in D0. The median parasite load for *S. mansoni* was 36 (12–108) epg, for *Ascaris lumbricoides*, it was 7425 (1452–24,810) epg, and for *Trichuris trichiura*, it was 420 (120–1236) epg, as shown in Table 2.

**Figure 1:**
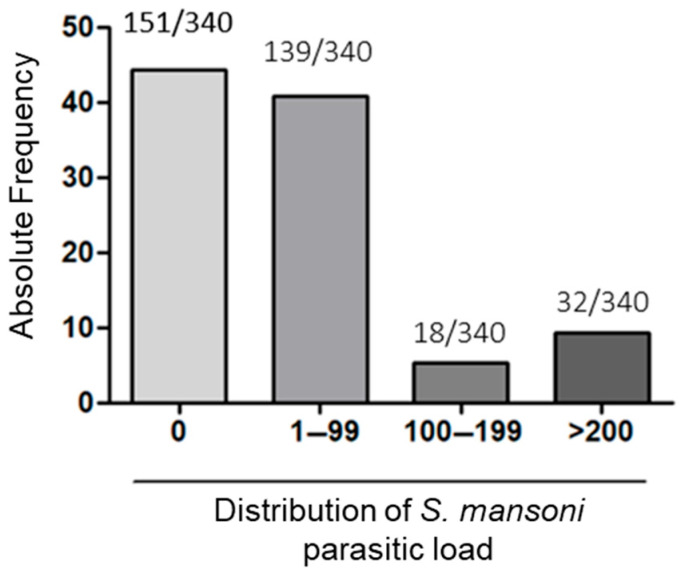
Distribution of the general study population according to *Schistosoma mansoni* infection status and parasite load. Percentage of the parasitic load referring to each group. High-load group (HL), medium-load group (MC), low-load group (LL) and Negative for *Schistosoma mansoni* residents of the endemic area (Neg). Analysis was performed with n = 340 individuals.

The study population was classified according to the parasitic load for *S. mansoni*. Individuals with a parasitic load above 200 epg, between 100 and 199 epg, and between 1 and 99 epg were defined as high load (HL), medium load (MC), and low load (LL), respectively. Among the participants at D0, 9.4% of individuals had a high parasitic load, 40.9% had a low parasitic load, while 5.3% of the participants had a parasitic load between 100 and 199 epg. Finally, 44.4% of the total had negative results for *Schistosoma mansoni* (Figure 1).

**Figure 2:**
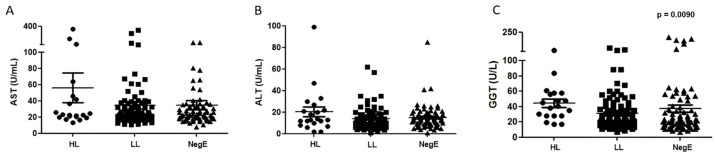
Serum concentrations of hepatic enzymes AST (**A**), ALT (**B**), and GGT (**C**) according to *Schistosoma mansoni* infection status and parasite load in the general study population Abbreviations: HL = high load; LL = low load; NegE = negative for *S. mansoni* infection; AST = Aspartate Aminotransferase; ALT = Alanine Aminotransferase; GGT = Gamma-Glutamyl Transferase. Note: Participants with helminth co-infections were not excluded from the analysis.

**Figure 3:**
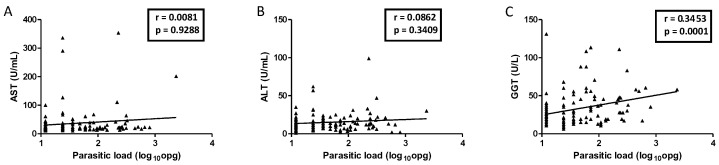
Spearman correlation between parasite load and serum concentrations of hepatic enzymes AST (**A**), ALT (**B**), and GGT (**C**) in *Schistosoma mansoni*-infected individuals from the general study population. The diagonal line was obtained from linear regression. Abbreviations: AST = Aspartate Aminotransferase; ALT = Alanine Aminotransferase; GGT = Gamma-Glutamyl Transferase. Note: Participants with helminth co-infections were not excluded from the analysis.

Of all the enzymes evaluated, GGT was the only one whose concentration varied among individuals with different parasite loads (*p* = 0.0090; Figure 2). A positive correlation (r = 0.3) was observed between the parasite load of *S. mansoni* and GGT levels (*p* = 0.0001; Figure 3). There was no statistically significant correlation between AST and ALT with parasite loads.

The lipidomic analysis was performed in 45 individuals, classified into three groups: high parasite load (HL), low parasite load (LL), and egg-negative endemic controls (NegE). Fifteen individuals were selected for each of these groups. Among the individuals who presented HL, those with the highest parasite load were selected.

The mean age of the HL group was 29.9 ± 20 years, that of the LL group was 20.7 ± 16.0 years, while the NegE group was 18.9 ± 14.6 years. Among the three groups, the HL group (33.3%) was the only one in which most individuals were not females. The median parasitic loads for the HL and LL groups were 312 (276–504) epg and 24 (12–24) epg, respectively. A higher body mass index (BMI) was observed in the HL group compared to the other groups (*p* = 0.0285) (Table 3).

Table 3 also presents the hepatic profile, in which it is possible to observe that, among the three groups, the HL group showed different values in all the evaluated biochemical parameters compared to the others (Table 3).

**Figure 4:**
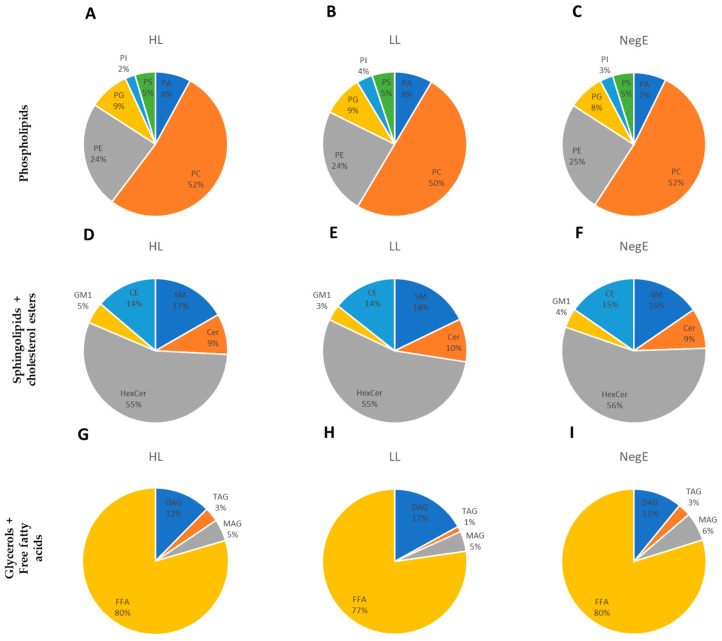
Relative abundance of lipid subclasses, including phospholipids (**A**–**C**), sphingolipids and cholesterol ester (**D**–**F**), and glycerides and free fatty acids (**G**–**I**). Show separately for each study group: high load (HL), low load (LL), and negative (NegE).

The lipidomic profile of the study population was determined by analyzing six subclasses of phospholipids, five of sphingolipids, and four of glycerides (which includes glycerols and free fatty acids (FFA)). The phospholipids that showed the highest relative abundance were phosphatidylcholine (PC) and phosphatidylethanolamine (PE), but no significant differences were observed among the high-load (HL), low-load (LL), and control (NegE) groups. Among the sphingolipids, the hexosylceramide (HexCer) subclass stood out the most, but also without any apparent difference. FFA was the most abundant subclass compared to TAGs and DAGs (Figure 4).

**Figure 5:**
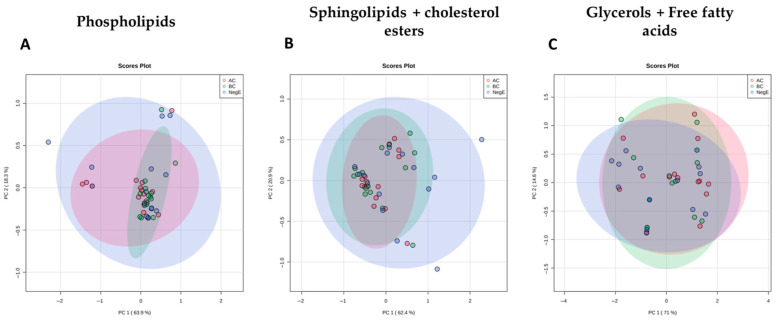
PCA analysis of lipid classes, namely, Phospholipid (**A**), Sphingolipid and cholesterol ester (**B**) and Glycerides and Fatty acid (**C**), showing the variation in the study groups.

Principal Component Analyses (PCA) were performed for each of the lipid families studied and showed a clear overlap of the different groups of individuals, suggesting that alterations in the levels of lipids in these families were not sufficient to distinguish individuals with different parasitic loads and uninfected individuals (Figure 5).

**Figure 6:**
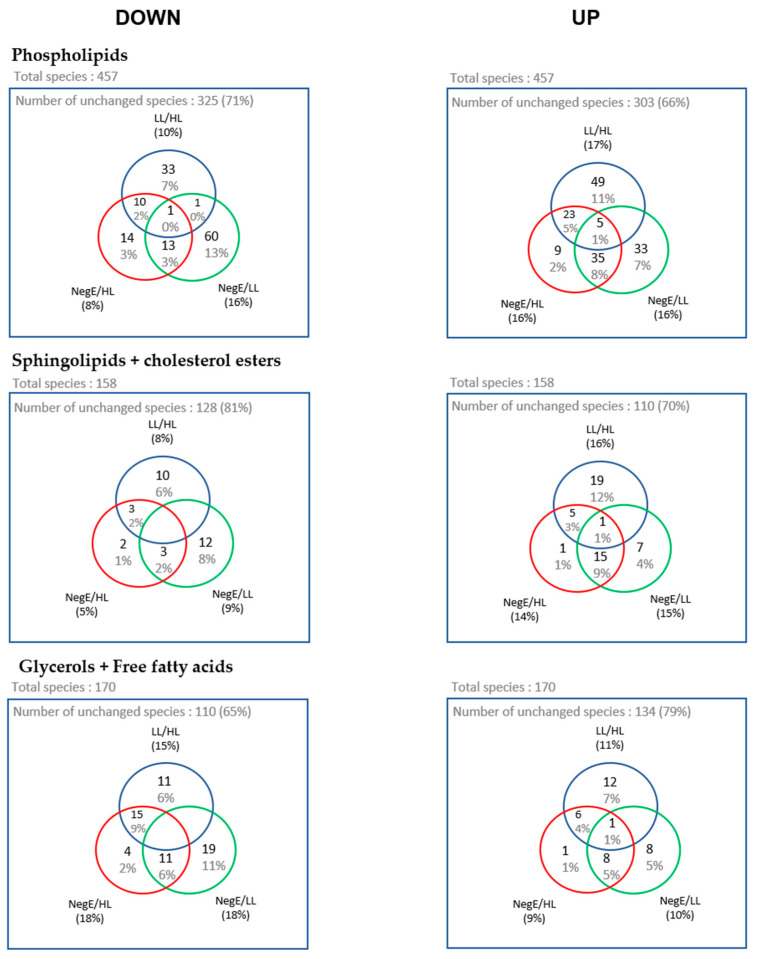
Venn diagram with the number of lipid species whose abundance varied significantly in the fold change analyses, and between the parasitic load groups. The data were separated between species that showed down and upregulation.

Next, we performed a fold change analysis to identify lipid species whose abundance varied between pairs: LL → HL, NegE → HL, and NegE → LL, allowing us to identify lipids that are increased (up) or decreased (down) between the groups. The number of lipids whose abundance varied between the evaluated groups is represented in the Venn diagrams in Figure 6.

From the Venn diagrams, we created heatmaps with the lipid species that were at the intersection between LL → HL, NegE → LL, considering that the pathology of schistosomiasis is directly associated with parasitic load [18]. Thus, this analysis represents the lipids that are systematically altered in the group of participants with high parasitic load, compared to individuals without schistosomiasis, or with low parasitic load, seeking to verify a relationship between the metabolic pathways associated with the pathology of schistosomiasis.

**Figure 7:**
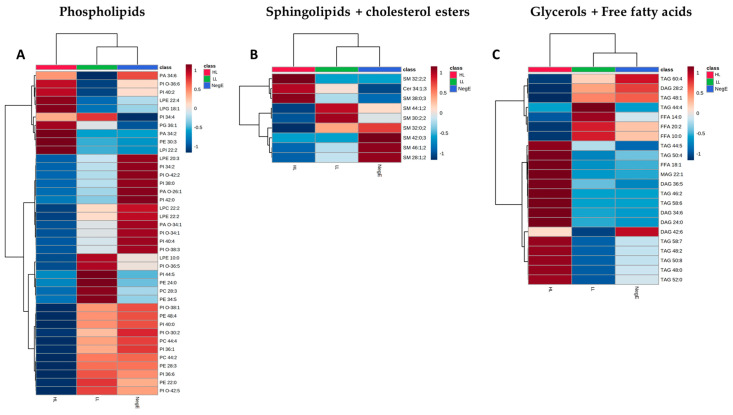
Heatmap of lipid species from the phospholipid (**A**), sphingolipid and cholesterol ester (**B**), and glyceride and free fatty acid (**C**) classes, which showed significant changes in fold change analyses when compared between the high-load (red), low-load (green), and negative (blue) groups. Expression is shown with dark blue (low expression) and dark red (high expression).

Based on the Venn diagrams, 39 phospholipid species, 9 sphingolipid species, and 22 glyceride species had their expression levels systematically altered in the HL group. The grouping of these lipid species is represented in the heatmaps of Figure 7. In Figure 7, it is possible to identify 21 phospholipids with lower concentrations in individuals with HL, when compared to the LL and NegE groups. It was also observed that 12 (57%) of these species belong to the PI subclass. Regarding sphingolipids and cholesterol ester, no variation pattern was observed in the concentrations of lipid species that significantly differentiated the groups (Figure 7B). In Figure 7C, 14 lipid species exhibited high concentrations in the HL group when compared to the others; of these, nine (64%) belong to the TAG subclass.

## 3. Discussion

This study aimed to explore alterations in the serum lipidomic profile associated with *Schistosoma mansoni* infection under real-life epidemiological conditions in a hyperendemic area. By stratifying individuals based on *S. mansoni* parasite burden and including uninfected controls from the same population, we sought to identify lipid species potentially linked to infection intensity and hepatic involvement, while preserving the natural complexity of co-infections commonly found in such settings.

Alterations in hepatic enzymes and serum lipid profiles have been reported in individuals living in endemic areas for *S. mansoni*. Some hepatic enzymes, such as AST and ALT, are commonly used as indicators of liver injury, as they are present in large quantities in hepatocytes and may be released during hepatic inflammation or damage. In the context of *S. mansoni* infection, previous studies have described changes in transaminase levels during the inflammatory process, as well as increased gamma-glutamyltransferase (GGT) in individuals with hepatic granulomas [19,20].

The clinical alterations of schistosomiasis are associated with the location of the parasite, the mechanism used by the host’s immune system to combat the infection, and the parasite load. The main immunopathology is the formation of granulomas around the parasite eggs, which induces an intense immune response that results in clinical manifestations [3,21].

These complications cause alterations in serum hepatic levels. This study did not identify any significant association between serum transaminase concentrations and schistosomiasis parasite load. However, when correlating the parasite load of *S. mansoni* with the enzyme GGT, a positive correlation was observed. Some studies have shown elevated levels of GGT in patients with the hepatosplenic form of the disease [22,23,24]. Despite the good health data of the total participants, it is important to pay attention to the possible evolution of schistosomiasis in those with higher GGT levels.

Other factors interfere with serum GGT levels, such as alcohol. A study in patients with the hepatointestinal form of schistosomiasis and a healthy control group showed that GGT levels did not change significantly after alcohol consumption, suggesting that the mechanism for increased GGT is different in patients with *S. mansoni* and those with chronic alcohol consumption [25]. In addition to alcohol, fibrosis can also cause changes in GGT levels due to biliary compression caused by granuloma formation around the egg [20]. Therefore, further studies are needed to understand the mechanism responsible for increased GGT.

The severity of schistosomiasis varies according to the parasite load and can cause serious manifestations, mainly in long periods of infection and reinfection [26]. The liver, which is the organ responsible for the synthesis of proteins, carbohydrates, and lipids, is the most affected. When liver damage initially occurs, cells regenerate, and those that have undergone apoptosis or necrosis are replaced. However, when the damage is constant, the tissue recovery mechanism fails, and eventually, in an attempt to repair the damage, the organism enters a state characterized as fibrosis [27]. Thus, liver impairment during infection disrupts homeostasis, especially in its vital functions, such as lipid metabolism in the body [28].

In our study, we observed alterations in host lipid metabolism during *Schistosoma mansoni* infection, which may be linked to the parasite’s biological demands and host pathology. It is well-established that *S. mansoni* requires extensive lipid resources to support egg production and maintenance of its own metabolism [13]. Moreover, the parasite secretes various proteins and molecules capable of modulating host physiological pathways, potentially including lipid metabolism [29,30]. Alternatively, the observed lipid changes may also be a consequence of hepatic damage and inflammation caused by granulomatous responses to parasite eggs, which are known to disrupt liver function and lipid homeostasis [1]. These mechanisms are not mutually exclusive and together may explain the complex lipid alterations detected in infected hosts.

It is important to acknowledge that co-infections with other intestinal helminths, such as *Ascaris lumbricoides* and *Trichuris trichiura*, were present among participants, which may be viewed as a potential limitation of the study design. However, this condition accurately reflects the reality of populations living in endemic areas, where polyparasitism is highly prevalent due to poor sanitation and socioeconomic vulnerability [31]. In this context, we chose to focus on *Schistosoma mansoni* infection specifically, given its biological characteristics and clinical relevance. Unlike other helminths, the adult forms of *S. mansoni* reside in the mesenteric venous system, in direct contact with the host’s bloodstream [6]. This unique localization supports the rationale for investigating potential associations between *S. mansoni* infection and alterations in the host’s serum lipidomic profile.

Based on the lipidomic analysis, we categorize lipid species into three major families: phospholipids, sphingolipids plus cholesterol ester, glycerols, and free fatty acids. Each of these families could be classified into subclasses, with six subclasses for phospholipids, five subclasses for sphingolipids and cholesterol ester, and four subclasses for glycerols and free fatty acids. The method used enabled the identification of the relative abundance of each lipid species within its respective subclass. This approach was used to compare the lipid profiles of individuals with a high parasite load, who are more likely to have greater hepatic impairment, with the profiles of individuals with a low load or who are negative for the disease.

Initially, we identified a similar proportional distribution of lipid subclasses among individuals with high parasite load and those who were negative or had low parasite load. Phosphatidylcholine (PC) emerged as the most abundant phospholipid subclass in all groups, although no statistically significant differences in PC levels were observed between them. This pattern is consistent with a previous study involving eleven female patients infected with *S. mansoni*, in which PC was also the predominant subclass, despite differences in clinical context [32]. In addition, hexosylceramide (HexCer) also stood out among the subclasses of sphingolipids and cholesterol ester in all groups evaluated. A study conducted on patients infected with the hepatitis C virus (HCV), who had severe fibrosis, also showed a predominance of the HexCer subclass [33]. Regarding the family of glycerols and free fatty acids, our study demonstrated a relative predominance of free fatty acids (FFA), a lipid that is important for the production of *S. mansoni* eggs [34].

Although the subclasses PC, HexCer, and FFA were predominant in terms of total abundance across the dataset, a more detailed analysis revealed that phosphatidylinositol (PI) and triacylglycerol (TAG) exhibited the highest proportion of differentially regulated species. Despite their low overall abundance (3% for PI and 2% for TAG), these subclasses showed the greatest relative number of species with significant alterations when comparing the high-load group to the low-load and negative groups—57% of PI species and 64% of TAG species were differentially abundant.

Regarding phospholipid subclasses, it was observed that the PI species are decreased in the group of individuals with a high parasite load compared to the other groups. These data suggest that PI is being consumed more in individuals with a high parasite load. It has been shown that after the hydrolysis of PI, its fragments are capable of activating protein kinase C (PKC), which regulates many cellular functions of the worm, mainly in the cercaria and the adult worm, one of which is the maintenance of the integrity of the tegument and consequent persistence of the infection [35,36,37,38].

Another subclass that is important for the survival of worms is triacylglycerols (TAG) from the family of glycerol and free fatty acids. Fatty acids are a requirement for egg production. Adult females require energy and a quantity of fatty acids, thus having a greater daily absorption compared to the male. *S. mansoni* is not able to synthesize fatty acids, so they are taken from the environment and stored as TAG [34,39,40,41,42,43,44,45,46]. This corroborates the findings of the present study, where an increase in the concentrations of some TAG species was observed in individuals with a high load when compared to the other groups.

In general, our findings suggest that alterations in specific phosphatidylinositols (PI) and triacylglycerols (TAG) may be associated with high parasite burden and the persistence of *S. mansoni* infection. In particular, the increased abundance of PI species and reduced levels of certain TAGs observed in individuals with intense infection indicate a potential link to immune–metabolic disturbances. Although sample size limits definitive conclusions, these lipid species may serve as exploratory candidates for biomarkers of severe or progressive infection. Further studies with larger cohorts are needed to validate these associations and investigate their clinical relevance.

## 4. Materials and Methods

### 4.1. Study Population

This study is a cross-sectional analysis that is part of a larger, longitudinal project that was conducted in three villages in the rural area of the municipality of Conde—BA, located approximately 170 km from Salvador—BA, from 2018 to 2019. All residents of the villages were invited to participate in the study. A total of 340 individuals aged between 4 and 60 years, who completed the blood, stool, and urine collection stages and who completed the interviews, were included for parasite load assessment and determination of serum concentrations of liver enzymes.

For lipidomics analyses, from the 340 study participants, 45 individuals were selected and subdivided into three groups of 15, according to their *Schistosoma mansoni* infection status and parasite load: (1) individuals with a parasite load above 200 eggs per gram of feces (epg), defined as the “High load” (HL) group; (2) individuals with a parasite load between 1 and 99 epg, referred to as the “Low load” (LL) group; (3) individuals living in the endemic area with a negative Kato–Katz test result for *S. mansoni*, comprising the “Negative” (NegE) group. Participants in the LL group were randomly selected from eligible individuals within the defined parasite load range. For the HL group, the 15 individuals with the highest parasite loads were intentionally selected to ensure inclusion of participants with the most intense infections, under the assumption that they would be more likely to exhibit measurable metabolic alterations. Co-infection with other helminths was not an exclusion criterion, as polyparasitism is common in this population and reflects the real-world conditions of endemic settings. No additional exclusion criteria related to age, sex, or clinical condition were applied.

The diagnosis of *S. mansoni* infection was performed using the Kato–Katz method, from two slides of a single sample. The slides were prepared and evaluated at the Laboratory of Experimental Pathology (LAPEX), Gonçalo Moniz Institute (IGM)/FIOCRUZ-BA, and blood was collected for serum dosages and lipid evaluations. The serum samples were separated by centrifugation and stored appropriately.

After collection and diagnosis, all infected individuals from the Conde-BA area with helminths were treated with Albendazole (single dose, 400 mg for adults and children over 2 years), while those patients with schistosomiasis were treated with Praziquantel (50 mg/kg for adults and 60 mg/kg for children between 4 and 14 years), following the guidelines recommended by the Ministry of Health.

### 4.2. Evaluation of Liver Enzymes

Blood samples were collected in tubes with a separating gel and centrifuged, and approximately 1 mL of serum was obtained from each participant. The serum was used to measure aspartate aminotransferase (AST), alanine aminotransferase (ALT), and gamma-glutamyltransferase (GGT) for lipidomic analyses. In addition, blood was collected in tubes containing EDTA for complete blood counts.

All dosages were performed using a semi-automatic biochemistry analyzer model BIO-200 Bio-Plus^®^, available at Fiocruz—BA. Reagents from Bioclin^®^ Biochemistry Kits (Bioclin, Belo Horizonte, Brazil) were used, and they were provided free of charge through a specific company program for research incentives.

### 4.3. Lipidomic Analysis

Lipid extraction and lipidomic analysis of the 45 individuals selected for the study were performed at the platform of the Center of Excellence in Quantification and Identification of Lipids (CEQIL), Faculty of Pharmaceutical Sciences of Ribeirão Preto (FCFRP), Ribeirão Preto, SP. For extraction, 500 µL of methanol (MeOH), 10 µL of internal standard, and 250 µL of CHCl3 (Chloroform) were added to 200 µL of serum. The mixture was homogenized in a ball mill homogenizer for 5 min, then 250 µL of CHCl3 and 250 µL of Milli-Q water were added, homogenized for 5 min, and centrifuged for 5 min at 10,000 RPM and 4 °C to separate the phases. The lower phase of the tube was removed and stored, while 500 µL of CHCl_3_ was added to the upper phase, homogenized, and centrifuged. Finally, the lower phase of the tube was removed, and the remaining content was subjected to a drying step for one hour at 45 °C and stored at −20 °C.

The global lipidomics method was used based on analyses with a high-accuracy mass spectrometer coupled to a liquid chromatography system (Nexera-TripleTOF^®^ 5600+ (LC-MS/MS)) (Sciex, Foster City, CA, USA). After extraction, the samples were resuspended with 200 µL of solvent (mixture of Isopropanol, MeOH, acetonitrile, Milli-Q water, and ammonium formate) (Aldrich Chemistry, St. Louis, MO, USA), subjected to agitation for 10 min, centrifuged for 5 min at 10,000 RPM and 4 °C, and applied to the spectrophotometer.

In the lipidomic analysis, five classes of lipids were identified: phospholipids, sphingolipids, cholesterol esters, glycerols, and free fatty acids. Among the phospholipids, phosphatidic acids (PA), phosphatidylcholine (PC), phosphatidylethanolamine (PE), phosphatidylglycerol (PG), phosphatidylinositol (PI), and phosphatidylserine (PS) were evaluated. In the group of sphingolipids, five classes were analyzed: ceramide (CER), hexosylceramide (HexCer), sphingomyelin (SM), and gangliosides 1 (GM1). Finally, in the group of glycerides, diacylglycerol (DAG), monoacylglycerols (MAG), and triacylglycerols (TAG) were quantified. Cholesterol ester (CE) and free fatty acids (FFA) were also quantified.

### 4.4. Statistical Analyses

All data collected were stored in the REDCap^®^ system (Vanderbilt University, Nashville, TN, USA). The data were analyzed using GraphPad Prism 5 and Stata 11.0 software, as well as MetaboAnalyst 5.0 (https://www.metaboanalyst.ca/, accessed on 22 August 2022).

Mean, standard deviation, median, and interquartile range (25–75%) were described. Statistical tests were performed, such as a Student’s *t*-test and an Anova using GraphPad Prism 5 software for the analysis of liver enzymes and serum levels. Spearman correlation was performed for the correlation analyses between liver enzymes and parasite load.

In the lipidomic analysis, the chemical names of the lipid species were identified based on individual fragmentation patterns and with the help of the LIPID Metabolite and Pathways Strategy (LIPID MAPS) platform (http://www.lipidmaps.org/, accessed on 22 August 2022). Data quantification was performed by comparing the areas of the chromatograms of the precursor masses of the samples with the help of the PeakView program (Sciex) (https://sciex.com/, accessed on 22 August 2022).

In the MetaboAnalyst platform, for the execution of the principal component analysis (PCA), it was necessary to remove data that differed drastically from all others, known as outliers. A platform that calculates and highlights outliers was used. Regarding the data generated by the Venn diagram, all significant lipid species were selected from the fold change analysis that shows the relationship of parasite load groups between lipid classes, and thus, the data were interpreted by Stata 11.0, using the proportional Venn diagram.

### 4.5. Ethical Aspects

This work is part of a larger project, which is approved by the Research Ethics Committee (CEP) of the Gonçalo Moniz Institute, entitled “HEALTH EVALUATION OF A RURAL POPULATION IN THE STATE OF BAHIA” (CAAE No. 77287417.8.0000.0040), which has been ongoing since January 2018. During this period, D0 was executed, which is defined by the inclusion of participants after signing the informed consent form. In addition, stool and blood samples were collected, as well as interviews. The reading of the Kato–Katz slides was carried out by members of the research groups involved in the project at LAPEX IGM/FIOCRUZ-BA.

## 5. Conclusions

High *S. mansoni* parasite load was associated with alterations in serum levels of specific phosphatidylinositols (PI) and free fatty acids (FFA), suggesting a disruption in host lipid metabolism potentially linked to egg production and hepatic injury. While preliminary, these findings highlight PI and FFA species as promising candidates for future biomarker research and contribute to a better understanding of the metabolic impact of schistosomiasis.

## Figures and Tables

**Table 1:** Sociodemographic, hematological, and biochemical characteristics of the general study population living in an endemic area for *Schistosoma mansoni*.

Variables	General Population (n = 340)
Age—years [mean (±SD)]	28 (±18)
Sex—Women [% (n)]	57.06 (194)
Presence of piped water [% (n)]	99.15 (337)
Presence of septic tanks [% (n)]	63 (214)
Average household income—Up to 1 minimum wage ^1^ [% (n)]	84.62 (288)
**Hematological profile [median (IQR)]**	
Hemoglobin (g/dL)	12.70 (11.80–13.68)
Total leukocytes (/µL)	6710 (5223–8205)
Platelets (thousands/µL)	268 (224–320)
**Biochemical profile [median (IQR)]**	
AST (U/L)	22.37 (18.64–32.63)
ALT (U/L)	12 (9–18)
GGT (U/L)	23.66 (17.21–40.63)

^1^ Minimum wage equivalent to BRL 954 at the time of data collection (2018). Abbreviations: SD, Standard Deviation; IQR, Interquartile Range; AST, Aspartate Aminotransferase; ALT, Alanine Aminotransferase; GGT, Gamma-Glutamyl Transferase.

**Table 2:** Frequency and parasite load of intestinal helminth infections in the general study population.

Variables	General Population (n = 340)
***Schistosoma mansoni***	
Infection prevalence [% (n)]	55.59 (189)
Parasite load (epg) [median (IQR)]	36 (12–108)
***Ascaris lumbricoides***	
Infection prevalence [% (n)]	40.29 (137)
Parasite load (epg) [median (IQR)]	7425 (1452–24,810)
***Trichuris trichiura***	
Infection prevalence [% (n)]	53.82 (183)
Parasite load (epg) [median (IQR)]	420 (120–1236)

Abbreviations: IQR, Interquartile Range; epg, eggs per gram.

**Table 3:** General characteristics of the study groups selected for lipidomic analysis (n = 45), according to *Schistosoma mansoni* infection status and parasite load.

Variables	HL (n = 15)	LL (n = 15)	NegE (n = 15)	*p* ^1^
Age (mean ± SD)	29.9 ± 20.0	20.7 ± 16.0	18.9 ± 14.6	>0.05
Women [% (n/total n)]	33.3 (5/15)	60.0 (9/15)	46.7 (7/15)	>0.05
*Schistosoma mansoni*				
Parasite load in epg [median (IQR)]	312 (276–504)	24 (12–24)	0	<0.0001
Biochemical profile [median (IQR)]				
AST (U/L)	19.7 (16.9–25.9)	22.0 (18.4–34.0)	22.0 (19.8–26.3)	>0.05
ALT (U/L)	16.0 (9.0–20.3)	12.0 (7.5–17.5)	12.0 (9.0–17.0)	>0.05
GGT (U/L)	27.9 (22.5–38.8)	23.6 (15.0–37.8)	21.5 (17.3–27.9)	>0.05

^1^ Mean age was compared using one-way ANOVA. The frequency of women was compared using the chi-square test. Median values of parasitic load and biochemical data were compared using the Kruskal–Wallis test. Abbreviations: HL, high load; LL, low load; NegE, negative for *S. mansoni* infection; SD, Standard Deviation; IQR, Interquartile Range; epg, eggs per gram; AST, Aspartate Aminotransferase; ALT, Alanine Aminotransferase; GGT, Gamma-Glutamyl Transferase.

## Data Availability

The data presented in this study are available on request from the corresponding author.
